# Differential regulation of SC1/PRDM4 and PRMT5 mediated protein arginine methylation by the nerve growth factor and the epidermal growth factor in PC12 cells^☆^

**DOI:** 10.1016/j.neulet.2013.06.051

**Published:** 2013-08-29

**Authors:** Alexandra Chittka

**Affiliations:** Wolfson Institute for Biomedical Research, University College London, London, WC1E 6BT, United Kingdom

**Keywords:** SC1, Schwann cell factor 1, PRMT, protein arginine methyltransferase, PRDM, positive regulatory domain protein, NGF, nerve growth factor, EGF, epidermal growth factor, NSC, neural stem cells, (H)MTase, (histone) methyltransferase, PRDM4, PRMT5, Histone arginine methylation, PC12 cells, NGF

## Abstract

During neuronal development, the neuroepithelial stem cells (NSCs) initially undergo proliferative divisions, later switching to neurogenic ones whereby one NSC and a post-mitotic neuron are generated. We recently showed that a member of the PRDM family of transcriptional regulators, PRDM4/SC1, recruits a type II protein arginine methyltransferase, PRMT5, to maintain the “stem-like” cellular state of the embryonic mouse cortical NSCs. However, little is known about the regulation of activity of this complex under proliferation- or differentiation-inducing growth conditions.

In the present work I investigate the regulation of SC1/PRMT5-mediated methylation activity in PC12 cells treated with EGF or NGF. I present evidence that NGF down-regulates SC1/PRMT5 methyltransferase (MTase) activity and that the reduction in SC1/PRMT5 MTase activity occurs mainly in the nucleus. I suggest that high levels of SC1/PRMT5 activity are associated with the proliferative state of the cells.

## Highlights

• SC1/PRMT5 histone methyltransferase activity is down-regulated by NGF, but not EGF.
• NGF reduces the nuclear, but not the cytosolic SC1/PRMT5-mediated HMTase activity.
• SC1 and PRMT5 are found in the nucleus and the cytosol of primary mouse neurons.

## 1 Introduction

During cortical development, the neuroepithelial stem cells initially proliferate to increase their numbers and later differentiate generating neurons and glia [20,23]. In part the decisions of the developing NSCs to continue proliferation or to commit to a differentiation programme are cell-intrinsic [14,15]. Additionally, extracellular growth factors regulate cell fate decisions of the precursor cells during development. Intriguingly, various growth factors bind to their cognate receptors to activate seemingly identical downstream pathway, leading to dramatically different cellular responses. For example, neurotrophins, e.g. the Nerve Growth Factor (NGF), activate receptor tyrosine kinases, but so do growth factors such as the Epidermal Growth Factor (EGF) [4]. However, the biological responses are either differentiation and cell cycle exit or proliferation, respectively [4].

Neurotrophins regulate many aspects of neuronal development via activation of two types of receptors, the low affinity p75 neurotrophin receptor, p75NTR, and a family of receptor tyrosine kinases, the Trk receptors, TrkA, TrkB and TrkC [24]. PC12 cells provide a convenient cellular system to investigate the differences in the signalling mechanisms of the neurotrophins and proliferation-inducing factors, e.g., EGF. PC12 cells express both the p75NTR and TrkA receptors and respond to NGF by differentiating into sympathetic-like neurons and to EGF or serum by proliferating [30]. The duration of ERK kinase activation is one of the critical parameters in distinguishing cellular response to either NGF or EGF in these cells [3,19,21].

We recently demonstrated that a p75NTR interacting protein, PRDM4/SC1 (referred to as SC1 henceforth), recruits a type II protein arginine methyltransferase, PRMT5, to direct histone arginine methylation in the neural precursor cells from the developing mouse cortex [7]. Moreover, one of the PRMT5-mediated histone modifications, namely histone H4 arginine 3 symmetric dimethylation (H4R3me2s) is a signature of the early proliferating neuroepithelium prior to the onset of neurogenesis [5]. Together these observations suggest that high levels of methyltransferase (MTase) activity of the SC1/PRMT5 complex may maintain the proliferative status of the NSCs. Recent investigations into the mechanisms responsible for growth- or differentiation-promoting activities identified PRMT5 as one of the critical modulators of EGF- or NGF-mediated biological responses in PC12 cells demonstrating that NGF reduces PRMT5 enzymatic activity [2]. I reasoned that SC1/PRMT5-mediated protein/histone arginine methylation may represent a subset of targets for PRMT5 methylation and that the activity of this complex may be differentially regulated by different types of growth factors, e.g., EGF and NGF in PC12 cells.

I demonstrate that NGF down-regulates SC1/PRMT5-mediated MTase activity consistent with the previously published observations that PRMT5 activity in PC12 cells is dampened by NGF [2]. Moreover, NGF-induced reduction in SC1/PRMT5-mediated MTase activity is confined to the nucleus, but not the cytosol of PC12 cells. EGF, however, sustains similar amounts of MTase activity by the complex in both cellular compartments. Finally, I show that both SC1 and PRMT5 are found in the nucleus and the cytosol of the early born primary mouse cortical neurons.

## 2 Materials and methods

### 2.1 Cell culture and transfections

PC12 cells were cultured in DMEM (Invitrogen) supplemented with 10% (v/v) horse serum (HS), 5% (v/v) foetal calf serum (FCS) and glutamine. For growth factor treatment the serum content was lowered to 1% HS. EGF (PeproTech) was used at 20 ng/ml, NGF (PeproTech) - at 50 ng/ml. Transfections were performed using Lipofectamine 2000 (Invitrogen) according to the manufacturer's instructions. 48 h post-transfection cells were treated with the indicated growth factors, lysed and processed for the histone methyltransferase activity (HMTase) as described [7].

### 2.2 Immunoprecipitation and methylation assays

PC12 cells were harvested in immunoprecipitation (IP) buffer (50mM Tris–HCl, pH7.4, 0.5% (v/v) Nonidet P-40, 300 mM NaCl) supplemented with the protease inhibitor cocktail (Sigma) and phosphatase inhibitor cocktails 1 and 2 (Sigma), processed as previously described and collected on protein A/G beads (Santa Cruz) [7]. After washing, beads with immunoprecipitated (IPed) proteins were processed for a radioactive in vitro HMTase assay as previously described using a mixture of purified calf thymus histones (Roche Applied Science) [7]. Products of HMTase reactions were separated by SDS-PAGE, transferred onto polyvinylidene difluoride (PVDF) membranes (Millipore) and visualized using fluorography.

### 2.3 Subcellular fractionation of the cells and histone extraction

PC12 cells treated as specified were harvested and processed for cytoplasmic and nuclear fractionation. Cells were rinsed in cold PBS on ice, scraped off the plates, resuspended in Hypotonic Buffer A (HBA) (10 mM HEPES-K^+^ pH7.5, 10 mM KCl, 1.5 mM MgCl_2_, 0.5 DTT) supplemented with protease (Sigma) and phosphatase inhibitors (Sigma) and pelleted by centrifugation at 1000 rpm for 5 min at 4 °C. HBA was supplemented with NP40 to a final concentration of 0.5% and added to the pellet to lyse the cells for 10 minutes are 4 °C. Nuclei were pelleted at 3000 rpm for 2 min at 4 °C and the supernatants collected as the cytosolic fraction, aliquoted and stored at −80 °C. Pellets containing nuclei were washed with HBA without NP40, followed by resuspension in buffer C (20 mM HEPES-K^+^ pH 7.9, 420 mM NaCl, 0.2 mM EDTA, 1.5 mM MgCl_2_, 0.5 DTT, 25% Glycerol) supplemented with protease and phosphatase inhibitors. Nuclear proteins were extracted for 30 min on ice with regular vortexing. The suspension was centrifuged at 4 °C for 10 min and the supernatant containing nuclear proteins collected, aliquoted and stored at −80 °C or used for HMTase assays immediately.

Histones were extracted from PC12 cells as follows. Cells were washed twice in cold PBS, lysed in Triton extraction buffer (TEB: PBS, 0.5% Triton X 100 (v/v), 2 mM phenylmethylsulfonyl fluoride (PMSF), 0.02% (v/v) NaN_3_) for 10 min on ice, centrifuged at 2000 rpm for 10 min at 4 °C, the supernatants discarded, pellets washed again in half the volume of TEB and centrifuged as before. Pellets were resuspended in 0.2 N HCl and extracted overnight at 4 °C. Extracts were centrifuged at 2000 rpm for 10 min at 4 °C and the supernatants used for SDS-PAGE.

### 2.4 Western blotting, immunocytochemistry and antibodies used

Western blotting was performed in TBST buffer (100 mM Tris–HCl, pH 7.5, 150 mM NaCl, 0.1% (v/v) Tween 20). For IP's and Western blotting the following antibodies were used: anti-myc (Upstate, 1:1000 for Western blotting), anti-H3 (Abcam, 1:1000), anti-H4 (Abcam, 1:500), anti-H4R3me2s (Abcam, 1:1000), anti-H4R3me2a (asymmetric dimethyl, Active Motif, 1:1000), anti-TBP (Abcam 1:2000), anti-α-tubulin (Abcam, 1:5000). The following antibodies were used for immunocytochemistry: anti-TuJ1 (Sigma, 1:500), anti-PRMT5 (Upstate Biotech, 1:100), anti-SC1/PRDM4 (a gift from Pilar Perez and Moses Chao (1:40) and our own (1:100) [6,7]). For immunocytochemical detection of antigens, primary neural stem cells were processed as previously described [7]. Fluorescent images were collected using the Leica Microsystems SPE confocal microscope. The following secondary antibodies were used: goat anti-rabbit Alexa 488, goat anti-mouse Alexa 568 (Invitrogen).

### 2.5 Primary neural stem cell cultures

Primary cortical neural stem cells were isolated from time mated mouse E10.5 embryos according to a published protocol [7] and plated at a density of 2.5 × 10^5^ cells/13 mm on glass cover slips. NSCs were cultured in DMEM supplemented with 40 ng/ml Neurotrophin-3 (NT-3) (Pepro Tech), 0.25% FCS, B27 supplement, sodium pyruvate and glutamine (all from Invitrogen). Animal experiments were approved by the University College London local ethical committee and conformed to the UK Animals (Scientific Procedures) Act 1986. Project license number PPL 70/6697.

## 3 Results

### 3.1 NGF down-regulates SC1/PRMT5-mediated HMTase activity

**Fig. 1:**
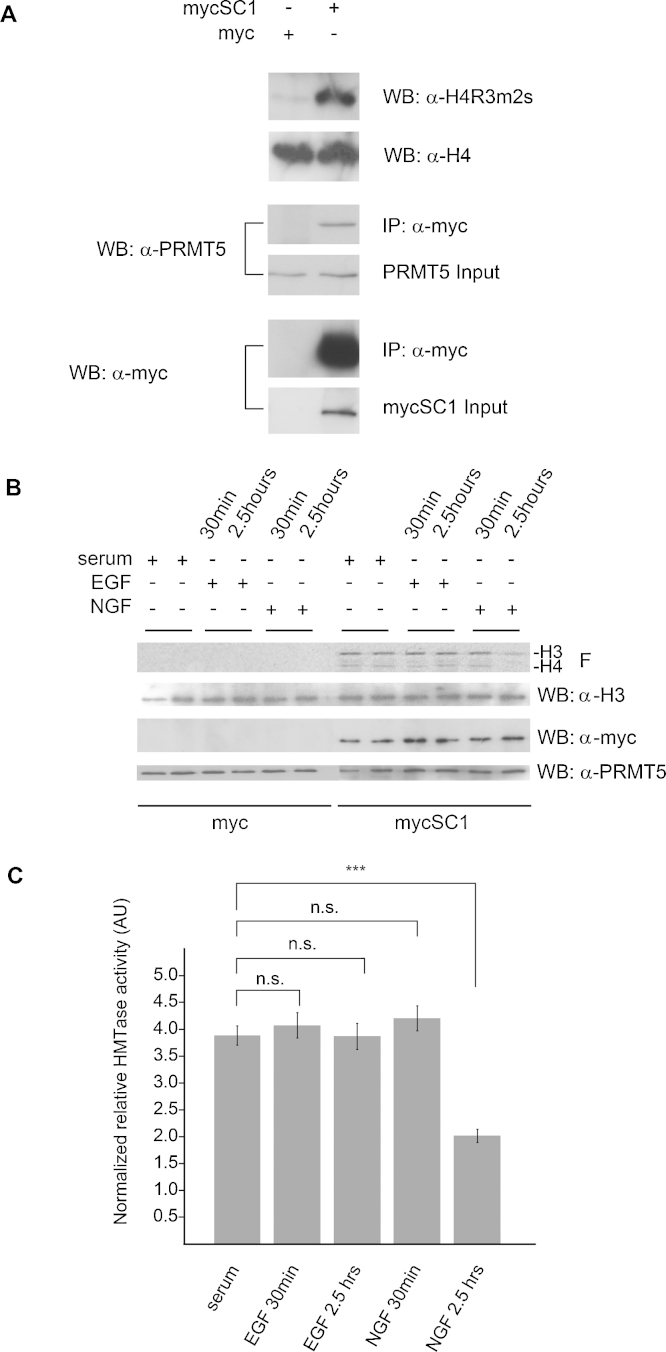
**NGF down-regulates SC1/PRMT5-mediated HMTase activity in PC12 cells**. (A) mycSC1 or Myc-tag alone were overexpressed in PC12 cells, the proteins IPed using anti-myc antibody and used for an in vitro HMTase assay with purified calf thymus histones. The products of HMTase reaction were probed with anti-H4R3me2s antibodies (top panel) and with anti-H4 antibodies to control for the histone loading (2^nd^ panel from the top). Input, IPed and co-iped proteins were detected by using indicated antibodies to detect endogenous PRMT5 or mycSC1 (middle two and bottom two panels, respectively). (B) Transfected PC12 cells were cultured with serum, EGF or NGF as indicated. Overexpressed myc and mycSC1 were IPed and used for in vitro radioactive HMTase assay with purified calf thymus histones. Top panel: HMTase reaction products; 2^nd^, 3^rd^ and bottom panels - Western blots probed with: anti-H3 antibody to control for the histone loading; anti-myc antibody to detect IPed mycSC1 protein, anti-PRMT5 antibody to detect PRMT5, respectively. (C) Quantification of normalized HMTase activity detected in PC12 cells grown in serum, EGF or NGF. Relative HMTase activity was normalized to PRMT5 levels in the lysates (means ± S.D., *n* = 3, *t* test, *P* < 0.001). AU: arbitrary units; n.s.: not significant; F: fluorogram; IP: immunoprecipitation; WB: Western blot.

We recently demonstrated that SC1 recruits the protein arginine methyltransferase, PRMT5, to mediate symmetric dimethylation of histone H4 on arginine 3 (H4R3me2s) [7] and showed that H4R3me2s modification mediated by PRMT5 is a “signature” of proliferating neuroepithelium in the developing mouse cortex [5]. These observations suggest that the SC1/PRMT5-mediated MTase activity may be subject to regulation by extracellular signals which instruct the NSCs to either proliferate or differentiate. To investigate whether SC1/PRMT5 MTase activity is regulated by differentiation or proliferation-inducing factors, I used PC12 cells which respond to NGF and EGF by differentiating into sympathetic-like neurons or by proliferating, respectively [30]. To determine if SC1 interacts with PRMT5 in PC12 cells, I transiently transfected PC12 cells with either Myc-tagged SC1- (mycSC1) or Myc-tag- overexpressing plasmids and immunoprecipitated overexpressed proteins 48 h post-transfection using anti-myc antibodies. A complex between mycSC1 and the endogenous PRMT5, but not between Myc-tag alone and PRMT5 was detected in PC12 cells (Fig. 1A, 3^rd^ panel from the top). Moreover, an in vitro HMTase assay using the immunoprecipitated proteins revealed an increase in the amount of H4R3me2s methylation consistent with the presence of PRMT5 in mycSC1 immunoprecipitated complex (Fig. 1A, top panel). To test whether EGF and NGF regulate SC1/PRMT5-mediated MTase activity in PC12 cells, the cells were transfected with mycSC1- or Myc-tag-overexpressing plasmids and treated with EGF or NGF 48 h post-transfection for indicated times. Following growth factor treatment mycSC1 or Myc-tag proteins were immunoprecipitated (Fig. 1B, 3^rd^ panel) and an in vitro radioactive HMTase assay with exogenously added purified calf thymus histone mixture was performed [7]. NGF treatment consistently lowered HMTase activity associated with SC1 (Fig. 1B, top panel, lane 2.5 h NGF, quantified in Fig. 1C). In PC12 cells SC1/PRMT5-associated HMTase activity had a higher preference for histone H3 than for histone H4 (Fig. 1B, top panel). PRMT5 substrate preference for H4 is in part mediated by a protein COPR5, whose absence leads to a higher preference for methylating H3R8 by PRMT5 [17] –a likely explanation for the preferred methylation of H3 observed in PC12 cells. Treatment of PC12 cells with EGF did not lead to any detectable change in the amount of SC1/PRMT5-mediated histone methylation (Fig. 1B, EGF lanes). Neither NGF nor EGF treatment affected the levels of PRMT5 protein (Fig. 1B, bottom panel), suggesting that NGF reduces SC1/PRMT5-mediated MTase activity.

### 3.2 NGF-dependent reduction in SC1/PRMT5-mediated MTase activity is confined to the nucleus

**Fig. 2:**
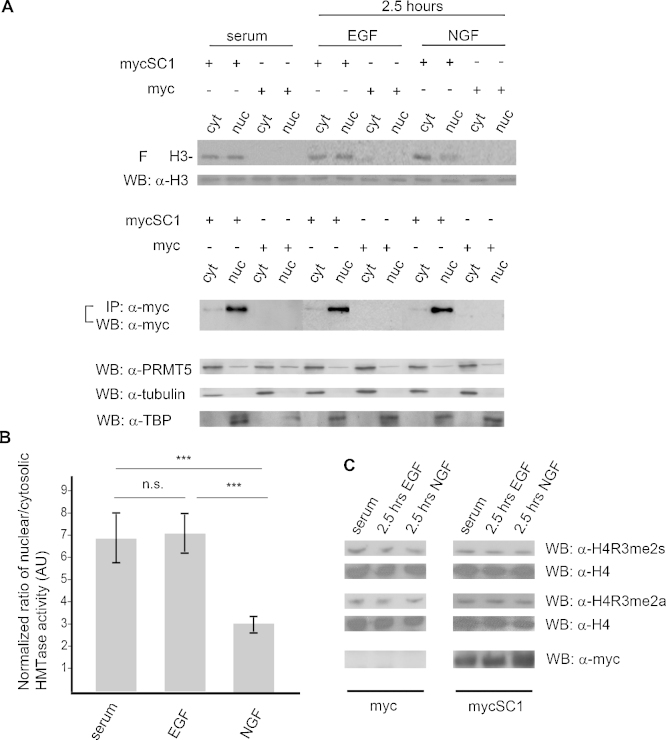
**NGF treatment of PC12 cells reduces SC1/PRMT5-mediated HMTase activity in the nucleus**. (A) PC12 cells were transfected as in Fig. 1, treated for 2.5 h with EGF or NGF 48 h post-transfection and fractionated into nuclear and cytosolic fractions. Overexpressed proteins were IPed from cellular fractions using anti-myc antibodies and used for an in vitro radioactive HMTase assay. Products of the HMTase reactions are shown in the fluorogram, top panel. Below is the Western blot using anti-H3 antibodies to control for histone loading. The middle panel shows IPed proteins from the nuclear and cytosolic fractions in the indicated reactions. The fourth panel shows endogenous PRMT5 in the lysates used for HMTase assay. Purity of subcellular fractions was evaluated by probing the blots with anti-tubulin and anti-TATA-binding protein (TBP) antibodies for cytosolic and nuclear fraction, respectively (bottom two panels). (B) Quantification of normalized HMTase activity ratio in the cytosolic/nuclear fractions of EGF or NGF treated PC12 cells. Relative HMTase activity was normalized to PRMT5 protein levels in the nuclear or cytosolic fraction (means ± S.D., *n* = 3, *t* test, *P* < 0.001). (C) Transfected PC12 cells were treated as in panel A, cellular histones extracted and probed with anti-H4R3me2s and H4R3me2a (asymmetric H4R3 dimethylation) antibodies; total H4 was detected with anti-H4 antibodies. Bottom panels show overexpressed mycSC1 protein from transfected PC12 cells. F: fluorogram; WB: Western blot; IP: immunoprecipitation; AU: arbitrary units.

Both SC1 and PRMT5 are found in the cytosol and in the nucleus [7,29]. PRMT5 methylates proteins in both compartments [2,29,31]. To investigate whether EGF and NGF regulate SC1/PRMT5-mediated MTase activity differentially within the cytosol and the nucleus, PC12 cells were transfected as described above and treated with either EGF or NGF for 2.5 h. Cells were lysed, separated into nuclear and cytosolic fractions, mycSC1 or Myc-tag proteins immunoprecipitated and an in vitro radioactive HMTase assay performed. NGF, but not EGF treatment induced a reduction in the amount of SC1/PRMT5-mediated HMTase activity within the nuclear compartment (Fig. 2A, top panel, compare NGF and EGF lanes, quantified in panel B).

NGF is known to increase asymmetrically dimethylated proteins [8]. We therefore investigated whether a change in the amount of endogenous histone arginine methylation (symmetric and asymmetric) is detectable in PC12 cells transfected and treated as described above. Histones extracted from PC12 cells following growth factor treatment, were subjected to a Western blot analysis with anti-H4R3me2s and anti-H4R3me2a antibodies. No significant change in the magnitude of H4R3me2s or H4R3me2a was detected in the EGF or NGF treated samples (Fig. 2C), suggesting that the histone modifications mediated by SC1/PRMT5 complex target a subset of specific gene loci which may not be detectable as an overall change in histone arginine methylation levels. Thus, NGF down-regulates SC1/PRMT5-mediated MTase activity within the nuclear compartment of PC12 cells without affecting total histone arginine methylation of H4R3.

### 3.3 SC1 and PRMT5 are found in the nucleus and the cytosol of the early-born neurons

**Fig. 3:**
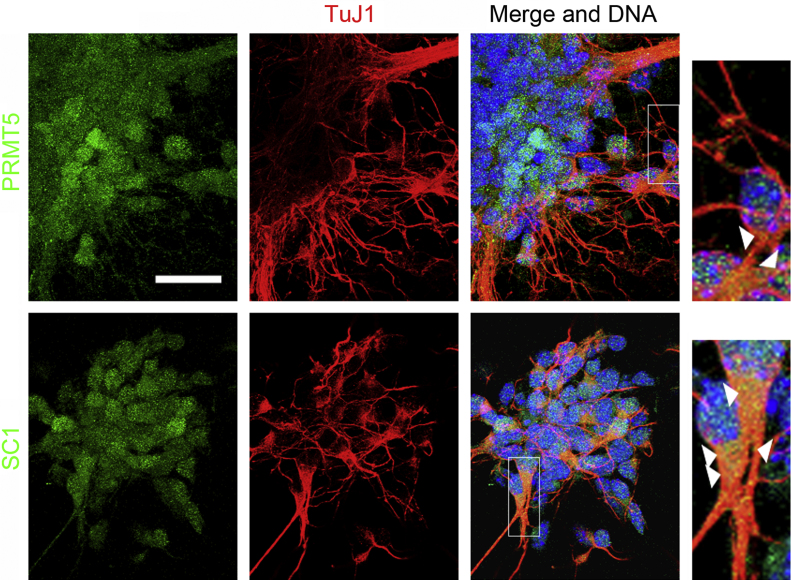
**SC1 and PRMT5 are found in the nucleus and the cytoplasm of early differentiating neurons**. NSCs isolated at E10.5 were cultured in the presence of NT3 to promote neurogenesis. After 3 days in vitro, the cells were immunolabelled with antibodies to visualize PRMT5, SC1 and TuJ1 to identify early-born neurons. Merged images showing Hoechst staining of DNA are shown on the right. Magnified images of the boxed areas from merged panels highlight (white arrowheads) the presence of SC1 and PRMT5 in both perinuclear and axonal parts of young neurons. Scale bar is 25 μm.

The observations, that NGF induced a selective reduction of the nuclear SC1/PRMT5-mediated MTase activity in PC12 cells, prompted me to investigate the subcellular distribution of SC1 and PRMT5 in differentiating neurons from the developing mouse cortex. Cortical NSCs isolated from E10.5 mouse embryos were cultured in NT3-containing media for 3 days to induce neurogenesis. Early-born neurons were detected by immunostaining cells with antibodies against Tuj1. Subcellular localization of SC1 and PRMT5 in early-born neurons was determined by immunostaining cells with appropriate antibodies. Both proteins were detected in the nucleus and cytosol of neurons (Fig. 3), suggesting that in differentiating neurons SC1/PRMT5 complex may mediate methylation of proteins in both subcellular compartments.

## 4 Discussion

Presented results demonstrate that SC1/PRMT5-mediated MTase activity is reduced by NGF, and sustained by EGF in PC12 cells. These observations are in line with the previously published report that NGF dampens PRMT5 activity in PC12 cells [2] and highlight the importance of protein/histone arginine methylation in directing cellular decisions to proliferate or differentiate [2,29]. Moreover, PRDM proteins are emerging as important regulators of developmental commitment decisions within the progenitor cells in multiple tissues, including the nervous system [1,9–13,16,18,25–28,31].

Our previous work identified PRMT5-mediated deposition of H4R3me2s modifications as a “signature” of proliferating embryonic cortical NSCs suggesting a role for PRMT5 in the control of NSC proliferation and “stemness” [5]. Moreover, we previously showed that silencing SC1 expression in proliferating NSCs induces precocious neurogenesis [7], suggesting that the SC1/PRMT5-mediated histone modifications may “write” part of the “histone code” for the proliferative cellular state of the NSCs. Therefore the observation that SC1/PRMT5-mediated MTase activity is down-regulated by NGF in PC12 cells is consistent with the idea that dampening the activity of this complex favours differentiation.

Another interesting observation is the selective, NGF-induced reduction of SC1/PRMT5-mediated MTase activity in the nucleus of PC12 cells. The decrease in the nuclear SC1/PRMT5 MTase activity is likely to result in a diminution of proteins symmetrically dimethylated by the complex within this compartment. Interestingly, histones may not be the only targets of NGF-dependent reduction of SC1/PRMT5-mediated protein methylation as no notable change in the overall H4R3me2s was observed in PC12 cells overexpressing SC1 and treated with NGF. Perhaps other nuclear proteins are also substrates of SC1/PRMT5-mediated symmetric arginine dimethylation down-regulated by NGF. This possibility is consistent with the previously published report demonstrating NGF-dependent increase in the amount of asymmetrically dimethylated proteins in the nucleus and our previous observations that asymmetric dimethylation of H4R3 is detected in the post-mitotic neurons of the developing cortex, but not in the proliferating NSCs where H4R3me2s prevails [5,8]. Thus, high activity of SC1/PRMT5-mediated symmetric protein arginine dimethylation within the nucleus appears to constitute part of the programme of post-translational protein modifications which may favour the proliferative cellular state.

The selective down-regulation of the nuclear SC1/PRMT5-mediated MTase activity by NGF induces a localized, specific response of the cells to different growth factors, suggesting that some of the decisions between proliferation and differentiation are regulated by the magnitude of symmetric protein arginine dimethylation within the nuclear compartment. Importantly, PRMT5 has been shown to control ES cell pluripotency by methylating the cytosolic pool of histone H2A in the embryonic stem (ES) cells [29]. Moreover, PRDM16 and 3 mono-methylate a cytosolic pool of histone H3 on lysine 9 to mediate their biological functions [22]. Therefore, selective regulation of the cytosolic and nuclear enzymatic activities of PRMT5 and PRDM proteins may be an emerging mechanism of cellular re-programming during development–a topic which remains to be explored in future studies.
